# Circulating granulocyte lifespan in compensated alcohol‐related cirrhosis: a pilot study

**DOI:** 10.14814/phy2.12836

**Published:** 2016-09-13

**Authors:** Jonathan R. Potts, Neda Farahi, Sarah Heard, Edwin R. Chilvers, Sumita Verma, Adrien M. Peters

**Affiliations:** ^1^ Department of Gastroenterology and Hepatology Brighton and Sussex University Hospitals NHS Trust Brighton United Kingdom; ^2^ Department of Medicine Brighton and Sussex Medical School Brighton United Kingdom; ^3^ Department of Medicine University of Cambridge School of Clinical Medicine Cambridge United Kingdom; ^4^ Department of Nuclear Medicine Cambridge University Hospitals NHS Foundation Trust Cambridge United Kingdom; ^5^ Division of Clinical and Laboratory Investigation Brighton and Sussex Medical School Brighton United Kingdom

**Keywords:** Alcohol‐related liver disease, cirrhosis, granulocyte, neutrophil

## Abstract

Although granulocyte dysfunction is known to occur in cirrhosis, in vivo studies of granulocyte lifespan have not previously been performed. The normal circulating granulocyte survival half‐time (*G *− *t*_½_), determined using indium‐111 (^111^In)‐radiolabeled granulocytes, is ~7 h. In this pilot study, we aimed to measure the in vivo *G *− *t*_½_ in compensated alcohol‐related cirrhosis. Sequential venous blood samples were obtained in abstinent subjects with alcohol‐related cirrhosis over 24 h post injection (PI) of minimally manipulated ^111^In‐radiolabeled autologous mixed leukocytes. Purified granulocytes were isolated from each sample using a magnetic microbead‐antibody technique positively selecting for the marker CD15. Granulocyte‐associated radioactivity was expressed relative to peak activity, plotted over time, and *G *− *t*_½_ estimated from data up to 12 h PI. This was compared with normal neutrophil half‐time (*N *− *t*_½_), determined using a similar method specifically selecting neutrophils in healthy controls at a collaborating center. Seven patients with cirrhosis (six male, aged 57.8 ± 9.4 years, all Child‐Pugh class A) and seven normal controls (three male, 64.4 ± 5.6 years) were studied. Peripheral blood neutrophil counts were similar in both groups (4.6 (3.5 − 5.5) × 10^9^/L vs. 2.8 (2.7 − 4.4) × 10^9^/L, respectively, *P *=* *0.277). *G *− *t*_½_ in cirrhosis was significantly lower than *N *− *t*_½_ in controls (2.7 ± 0.5 h vs. 4.4 ± 1.0 h, *P *=* *0.007). Transient rises in granulocyte and neutrophil‐associated activities occurred in four patients from each group, typically earlier in cirrhosis (4–6 h PI) than in controls (8–10 h), suggesting recirculation of radiolabeled cells released from an unidentified focus. Reduced in vivo granulocyte survival in compensated alcohol‐related cirrhosis is a novel finding and potentially another mechanism for immune dysfunction in chronic liver disease. Larger studies are needed to corroborate these pilot data and assess intravascular neutrophil residency in other disease etiologies.

## Introduction and Background

Advanced forms of alcohol‐related liver disease are associated with high rates of bacterial and fungal sepsis, which are a frequent cause of hospitalization and death in patients with cirrhosis (Verma et al. 2006). In part, this relates to defects in neutrophil function, including impaired phagocytic capacity and high resting oxidative burst (Mookerjee et al. 2007). More recently, neutrophil dysfunction has been shown to occur in those with compensated cirrhosis and to be transmissible to the neutrophils of healthy controls by incubation in plasma from cirrhotic subjects (Tritto et al. 2011). In vitro studies have shown increased rates of neutrophil apoptosis in decompensated versus compensated liver disease, mediated through increased capsase‐3 activity (Ramírez et al. 2004). This, coupled with hypersplenism, has been used to explain neutropenia in cirrhosis.

The normal in vivo circulating neutrophil lifespan is controversial, with a wide range of values depending upon the method of measurement. When determined from the sequential recovery of autologous radiolabeled granulocytes from peripheral blood, the normal intravascular half‐life (*t*_½_) is ~7 h (Saverymuttu et al. 1985). A much longer value of 5.4 days has recently been described using in vivo heavy water (^2^H_2_O) labeling (Pillay et al. 2010), although significant concerns exist regarding the validity of this method (Li et al. 2011; Tofts et al. 2011). Indium‐111 oxine (^111^In) is a gamma‐emitting radionuclide with a 67 h physical half‐life that preferentially labels neutrophils in a stable manner and is therefore ideally suited for in vivo study of neutrophil kinetics. Neutrophils constitute the majority of circulating granulocytes, the remainder comprising small numbers of eosinophils and basophils. Granulocytes are key to the innate immune response, although their lifespan in patients with compensated cirrhosis is currently unknown.

## Aims

In this pilot study, we aimed to determine the intravascular survival time of ^111^In‐radiolabeled granulocytes in subjects with compensated alcohol‐related cirrhosis.

## Patients and Methods

Subjects with compensated alcohol‐related cirrhosis (Child‐Pugh class A) were recruited from the outpatient liver clinic at our institution. Cirrhosis was diagnosed either from previous liver biopsy or the combination of clinical findings and compatible radiology (typically computed tomography showing an irregular liver margin and features of portal hypertension). In all cases, other causes of liver disease were diligently excluded. To avoid confounding effects from alcohol‐induced bone marrow toxicity, all had been abstinent from alcohol for ≥6 months prior to recruitment. In all instances, we sought to verify self‐reported abstinence by reference to primary and secondary care records and excluded those in whom there was uncertainty. For comparison, healthy controls without liver disease were studied at a collaborating center. All participants were ambulatory outpatients at the time of the study without clinical evidence of active or recent infection.

### Leukocyte radiolabeling protocol

All subjects underwent conventional indium‐111 (^111^In)‐labeled leukocyte scintigraphy. Autologous mixed leukocytes were radiolabeled in vitro under sterile conditions according to published guidelines (Roca et al. 2010), taking precautions at all stages to minimize ex vivo cell perturbation. Briefly, 45 mL of venous blood was mixed with the anticoagulant acid‐citrate‐dextrose. Erythrocytes were allowed to sediment over 45 min, aided by the addition of 1% methylcellulose. A leukocyte‐rich, platelet‐depleted cell pellet was obtained by centrifugation of the supernatant and washed once with normal saline. The cells were resuspended in saline and incubated with approximately 25 MBq ^111^In‐oxine for 15 min, after which radiolabeling was terminated by the addition of autologous platelet‐poor plasma. The radiolabeled leukocytes were pelleted, the supernatant aspirated, and cell‐associated and unbound radioactivity measured to calculate the radiolabeling efficiency. Radiolabeled mixed leukocytes were resuspended in a further 3 mL platelet‐poor plasma and injected intravenously. The administered radioactivity was ~20 MBq.

Leukocyte labeling in normal controls recruited at the collaborating center was performed using ^111^In‐tropolone, an alternative ligand to oxine, although the labeling procedures were otherwise identical.

### Measurement of intravascular granulocyte and neutrophil residency time

#### Granulocytes

Sequential peripheral venous blood samples were obtained between 30 min and 10 h postinjection (PI) of ^111^In‐radiolabeled mixed leukocytes and again between 20–25 h PI. Purified granulocytes were separated from each whole‐blood sample using a magnetic microbead‐based antibody technique positively selecting for the granulocyte‐specific antigen CD15 (autoMACS, Miltenyi Biotec, Bergisch, Germany) (Zahler et al. 1997). The CD15‐associated radioactivity was measured using a *γ* counter (WIZARD 1480, PerkinElmer, MA) and expressed relative to the number of granulocytes per sample, determined using a hemocytometer. These values were expressed as a percentage of the peak value in each subject and plotted over time. Circulating granulocyte survival half‐life (*G *− *t*_½_) was calculated from the gradient of an exponential fitted to the data points acquired up to 12 h PI.

#### Neutrophils

In normal controls studied at the collaborating center, purified neutrophils were isolated from peripheral blood samples obtained up to 24 h PI using a similar negative selection antibody‐microbead technique, specifically selecting for neutrophils (RoboSep, StemCell Technologies, Vancouver, Canada). The normal neutrophil half‐life (*N *− *t*_½_) was determined in the same manner as *G *− *t*_½_.

### Statistical analysis

Data are presented as mean ± standard deviation, median (interquartile range), or number (%) and all reported *P*‐values are two‐tailed. Quantitative variables were compared using Student's *t*‐test or analysis of variance (ANOVA) and the Mann–Whitney *U*‐test or Kruskal–Wallis test for parametric and nonparametric data, respectively.

The study received external ethical approval and all participants gave informed written consent.

## Results

### Patient characteristics

Seven patients with cirrhosis and seven normal controls were studied. One had undergone previous liver biopsy and in the remaining six the diagnosis rested on clinical and radiological grounds. Compared with normal controls, subjects with cirrhosis were younger and a greater proportion were male, although these differences were not statistically significant (Table 1). Total peripheral leukocyte, neutrophil, and platelet counts were similar in both groups. All patients with cirrhosis were Child‐Pugh class A (Child‐Pugh score 6 in one case and 5 in all other subjects) and the stated median duration of abstinence from alcohol was 18 (11–84) months. Five (71.4%) had previously been admitted with episodes of hepatic decompensation, (severe alcoholic hepatitis, *n* = 3; variceal hemorrhage, *n* = 1 and refractory ascites, *n* = 1. All had radiological evidence of cirrhosis (irregular nodular liver margin), and in addition, two had splenomegaly, the maximum spleen size being 12.7 cm.

**Table 1:** Patient characteristics

	Compensated cirrhosis (*n* = 7)	Normal controls (*n* = 7)	*P*
Age (years)	57.8 ± 9.4	64.4 ± 5.6	0.137
Male gender	6 (85.7%)	3 (42.9%)	0.266
MELD score	9 (7.5–9.2)	–	–
Leucocyte count (×10^9^/L)	7.0 (7.0–9.2)	6.8 (4.9–7.4)	0.275
Neutrophil count (×10^9^/L)	4.6 (3.5–5.5)	2.8 (2.7–4.4)	0.277
Platelet count (×10^9^/L)	193 (89–256)	223 (188–260)	0.277

Data are presented as mean ± standard deviation, median (IQR) or number (%). MELD, modified end‐stage liver disease score.

Normal values: leucocyte count 4–11 × 10^9^/L, neutrophil count 2–7.5 × 10^9^/L, platelet count 150–450 × 10^9^/L.

### Intravascular granulocyte and neutrophil lifespan

**Figure 1:**
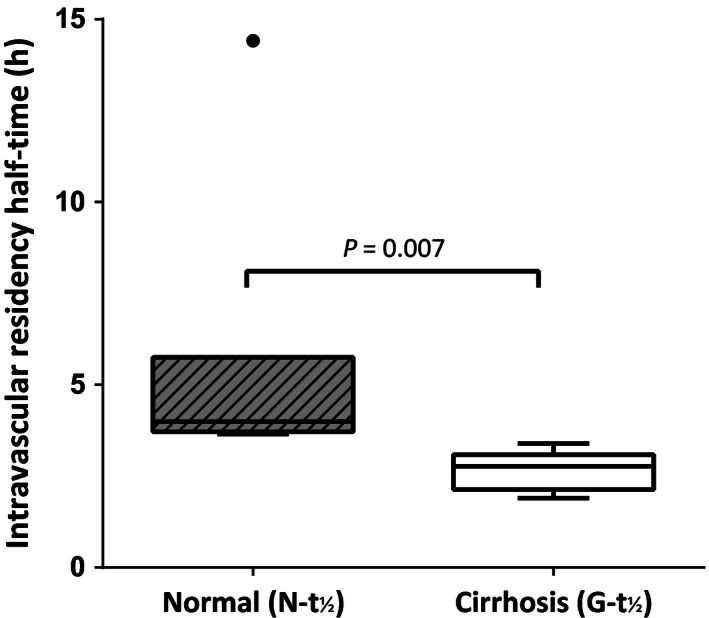
Comparison of intravascular granulocyte survival half‐time (*G *− *t*_½_) in alcohol‐related cirrhosis versus normal neutrophil half‐time (*N *− *t*_½_). Circulating granulocyte half‐lives in cirrhosis were significantly lower than normal neutrophil survival.

Mean normal circulating *N *− *t*_½_ determined from six healthy controls was 4.4 ± 1.0 h. One normal control in whom *N *− *t*_½_ was 14.4 h was deemed to be an outlier and excluded from the analysis. Mean *G *− *t*_½_ in cirrhosis was significantly shorter than the normal *N* − *t*_½_ (2.7 ± 0.5 h, *P = *0.007) (Fig. 1). *G *− *t*_½_ or *N *− *t*_½_ was unrelated to the peripheral neutrophil count (*ρ* = −0.249, *P *=* *0.412).

**Figure 2:**
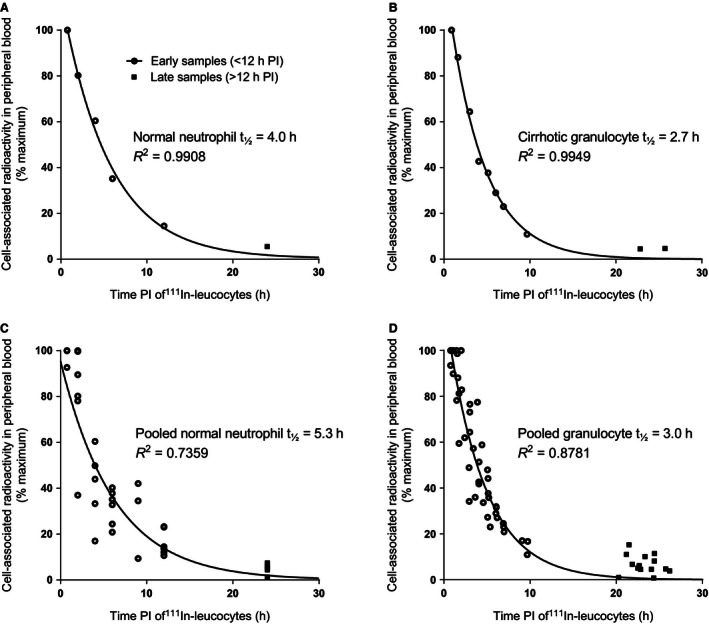
Clearance curves of ^111^In‐radiolabeled leukocytes from peripheral blood over time. Purified granulocytes or neutrophils were separated from peripheral venous blood samples obtained over 24 h postinjection (PI) of ^111^In‐radiolabeled mixed leukocytes. Corresponding rate constants and half‐lives were determined from curves fitted to data up to 12 h (PI). Example peripheral blood clearance curves of ^111^In‐labeled neutrophils in a normal control (A) and ^111^In‐labeled granulocytes in patient with compensated alcohol‐related cirrhosis (B). Pooled peripheral blood neutrophil recovery values in six normal controls (C) and granulocyte recovery in seven patients with compensated alcohol‐related cirrhosis (D).

Figure 2 shows example blood clearance curves in a normal control (A) and patient with compensated cirrhosis (B). Cell‐associated radioactivity up to 12 h PI followed a monoexponential decay function. Pooled recovery values up to 12 h PI in normal controls (Fig. 2C) displayed a greater spread than in cirrhosis (Fig. 2D), giving rise to accordingly lower *R* ^2^ values (0.74 vs. 0.88, respectively). In both groups, the intravascular half‐life determined from the exponential fit of pooled recovery values was similar to the mean of individual lifespan measurements (*G *− *t*_½_ in cirrhosis 3.0 h vs. normal *N *− *t*_½_ 5.3 h). Normal neutrophil recovery values after 20 h PI lay close to the extrapolated exponential generated from measurements up to 12 h PI. However, in cirrhosis, granulocyte‐associated radioactivity in later peripheral blood samples was higher than that expected from the earlier data. In 13 of 14 late samples (92.9%) obtained more than 20 h PI, CD15‐associated radioactivity was greater than that anticipated from the extrapolated exponential function generated using data up to 12 h PI (Fig. 2D).

**Figure 3:**
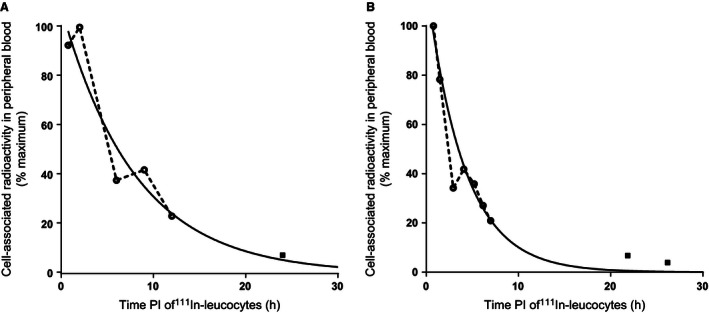
Example peripheral blood clearance curves in normal control (A) and cirrhosis (B) exhibiting transient rises in cell‐associated radioactivity, likely to represent re‐circulation of labelled neutrophils and granulocytes into the systemic circulation from as yet unidentified sites of margination.

Transient rises in neutrophil‐associated and CD15‐assoicated activities were observed in four normal controls and four patients with cirrhosis, respectively (example recovery curves shown in Fig. 3). These transient rises typically occurred later in normal subjects (~8–10 h PI) compared to those with cirrhosis (~4–6 h PI), commensurate with the shorter *G *− *t*_½_ in cirrhosis (Fig. 3).

## Discussion

Although defects in neutrophil function have been reported in various forms of chronic and acute‐on‐chronic liver disease, this pilot study is the first to report the in vivo measurement of granulocyte lifespan in cirrhosis. A number of findings are noteworthy. Firstly, normal intravascular neutrophil residency time determined from the recovered neutrophil fraction of radiolabeled mixed leukocytes was shorter than previously reported (~5 h vs. ~7 h) (Saverymuttu et al. 1985). Previous similar studies have radiolabeled purified neutrophils, a process that requires substantial ex vivo cell manipulation (Saverymuttu et al. 1985), which risks activating cells and consequently altering their in vivo behavior. The method utilized in this study minimized ex vivo cell perturbation during radiolabeling and isolated pure cell lines from the whole‐blood samples used in the measurement of radiolabeled neutrophil or granulocyte recovery. Furthermore, we determined the *t*_½_ of granulocytes and neutrophils using samples of whole‐blood obtained over a longer period (up to 12 h rather than 5 h in previous studies) (Saverymuttu et al. 1985).

Secondly, mean granulocyte lifespan in compensated alcohol‐related cirrhosis was significantly shorter than the normal neutrophil lifespan. This is despite the presence of small numbers of radiolabeled eosinophils selected in CD15‐positive samples, which, through their longer lifespan in blood (Farahi et al. 2012), would be expected to marginally prolong total granulocyte residency compared to pure neutrophil measurement. Suppressed granulocyte lifespan in cirrhosis is consistent with existing in vitro data suggesting increased frequency of neutrophil apoptosis in chronic liver disease (Ramírez et al. 2004).

A transient increase in the time courses of neutrophil and granulocyte‐associated activities was observed in normal controls and those with alcohol‐related cirrhosis (Fig. 3). These findings suggest recirculation of radiolabeled cells into the circulating granulocyte pool from sites of margination, resonating with recently reported findings using ^111^In‐labeled eosinophils (Farahi et al. 2012). The source(s) of recirculating granulocytes remain unknown and warrants further study with dynamic gamma camera imaging over carefully selected time points.

A “tail” in granulocyte recovery data was observed in alcohol‐related cirrhosis, likely reflect eosinophils isolated alongside neutrophils using CD15‐positive selection. Eosinophils have been shown to have a longer intravascular residency time than neutrophils (~25 h) (Farahi et al. 2012). Eosinophils constituted just 2.8 ± 1.2% of total granulocytes in these individuals and are therefore unlikely to substantially affect recovery values obtained up to 12 h PI. However, as a consequence of their long intravascular lifespan, over time, eosinophils form a greater proportion of residual circulating radiolabeled cells. The greater proportion of radiolabeled eosinophils in samples obtained after 20 h PI is likely to account for the prolonged curve seen in CD15‐positive separations in cirrhosis, not observed with purified neutrophil separations in normal controls.

Limitations inherent in the study methodology include the use of differing cell separation techniques in cirrhosis and normal controls due to the recruitment of subjects in two separate centers. However, since neutrophils comprise the vast majority of granulocytes (95.3 ± 2.1% in those with cirrhosis), the comparison between *N *− *t*_½_ and *G *− *t*_½_ appears to be scientifically appropriate. By calculating *G *− *t*_½_ from samples up to 12 h PI, the effect of radiolabeled eosinophils within CD15‐positive samples is minimized. It is possible that the shorter *G *− *t*_½_ we identified in cirrhosis relates in part to the differences either in leukocyte radiolabeling or cell selection techniques. Our method relies upon rapid and even distribution of radiolabeled cells between circulating and marginating granulocyte pools, and upon a steady rate of mature granulocyte release from the bone marrow during the period of measurement. Men were over‐represented in the cirrhotic group, consistent with the male predominance of those affected by alcohol‐related liver disease. However, we did not identify any difference in granulocyte or neutrophil residency according to gender (*P *=* *0.819). Finally, the sample sizes were comparatively small and would therefore benefit from further studies to corroborate the findings in a larger cohort.

We attempted to measure *G *− *t*_½_ using this technique in a further cohort with decompensated liver disease in the setting of severe alcoholic hepatitis (*n* = 3, data not shown). These individuals exhibited significant variability in peripheral blood neutrophil counts postradiolabeled leukocyte administration. Attempts to correct radiolabeled cell recovery data for changes in the peripheral neutrophil count generated radically differing values and hence we were unable to determine *G *− *t*_½_ in these subjects with any degree of confidence. However, future research with more refined techniques and a larger sample size may enable measurement of granulocyte lifespan in other forms of chronic liver disease, as well as acute and acute‐on‐chronic liver failure.

In conclusion, in this pilot study, we have shown for the first time that the intravascular granulocyte lifespan is suppressed in compensated alcohol‐related cirrhosis. This is of potential significance given the existing evidence for neutrophil dysfunction and resulting susceptibility to infection in cirrhosis. Infection is a frequent trigger of hepatic decompensation, often culminating in acute‐on‐chronic liver failure, and remains an important predictor of in‐hospital mortality. We identified the intravascular granulocyte lifespan in abstinent subjects with compensated alcohol‐related cirrhosis to be substantially lower than both normal controls and the previous reported normal circulating survival half‐time for ^111^In‐labeled granulocytes (Saverymuttu et al. 1985). The delayed recirculation phenomena observed in both normal individuals and those with cirrhosis are novel findings and warrant further study to determine the foci from which radiolabeled cells are released into the circulation.

## Conflict of Interest

SV: Advisory committees or review panels: Janssen; Grant/research support: Gilead, BMS, Janssen, Abbvie, Dunhill Medical Trust, National Institute of Health Research. JRP, NF, SH, ERC, AMP: None.
